# Plant Growth-Promoting Rhizobacteria for Cannabis Production: Yield, Cannabinoid Profile and Disease Resistance

**DOI:** 10.3389/fmicb.2019.01761

**Published:** 2019-08-08

**Authors:** Dongmei Lyu, Rachel Backer, W. George Robinson, Donald L. Smith

**Affiliations:** ^1^Crop Physiology Laboratory, Department of Plant Science, McGill University, Montreal, QC, Canada; ^2^Ravenquest Biomed, Inc., Vancouver, BC, Canada

**Keywords:** cannabis, cannabinoids, plant-growth promoting rhizobacteria, powdery mildew, biological control

## Abstract

Legal *Cannabis* production is now experiencing growing consumer demand due to changing legislation around the world. However, because of heavy restrictions on cannabis cultivation over the past century, little scientific research has been conducted on this crop, in particular around use of members of the phytomicrobiome to improve crop yields. Recent developments in the field of plant science have demonstrated that application of microbes, isolated from the rhizosphere, have enormous potential to improve yields, in particular under stressful growing conditions. This perspective carefully examines the potential for plant growth-promoting rhizobacteria (PGPR) to improve marijuana and hemp yield and quality. It then explores the potential use of PGPR for biological control of plant pathogens, which is particularly interesting given the stringent regulation of pesticide residues on this crop. As an industry-relevant example, biocontrol of powdery mildew, a common and deleterious pathogen affecting cannabis production, is assessed. Finally, two PGPR in genera frequently associated with higher plants (*Pseudomonas* and *Bacillus*) were selected as case studies for the potential effects on growth promotion and disease biocontrol in commercial cannabis production.

## Introduction

Cannabis production is drawing widespread attention because it can be used as food, fiber, medicine, and a recreational drug (Jiang et al., 2006; Kostic et al., 2008). The specific application and value is largely based on the concentration and composition of cannabinoids in cannabis plants (Sawler et al., 2015). The demand for cannabis is increasing as medical cannabis and cannabis production have been legalized in countries such as Colombia, Mexico, and Canada (Schuermeyer et al., 2014).

In medical cannabis production, the female plant is more desirable than the male for production of cannabinoids, due to higher flower biomass and cannabinoid levels (Potter, 2014). In commercial production, plants are propagated as cuttings from mother plants to produce genetically identical daughter plants to maintain population of desirable genotypes (Potter, 2014). Studies have attempted to determine which elements of cultivation and genetics contribute to cannabis yield and cannabinoid levels/composition. Cannabis yield is influenced by light intensity and plant density (Toonen et al., 2006; Vanhove et al., 2012; Backer et al., 2019). However, little research has been conducted regarding the response of yield and cannabinoid levels/composition to the application of plant-growth promoting rhizobacteria (PGPR), although research has already demonstrated the important role of PGPR on the production of many other crop species (Mabood et al., 2014; Smith et al., 2015). For example, the application of PGPR to plant roots can stimulate crop growth by providing mineral nutrition to plants. PGPR can also improve crop tolerance to abiotic stresses (e.g., drought and salinity) and biotic stress (e.g., plant pathogens) (Yan et al., 2016; Takishita et al., 2018).

Exploitation of PGPR from the phytomicrobiome (plant microbiome) will play an important role in industrial cannabis production, and there is a clear need to better understand the relationship between the phytomicrobiome and cannabis yield, cannabinoid levels/composition and disease resistance. This perspective summarizes knowledge about factors that contribute to cannabis yield and secondary metabolite biosynthesis. In addition, we examine the potential role of PGPR, with a focus on two widely prevalent genera (*Pseudomonas* and *Bacillus*), in achieving high yields, desirable cannabinoid profiles, and disease resistance in cannabis.

## Strategies to Increase Cannabis Yield and Quality

To achieve optimal quality for medical use, indoor marijuana cultivation aims to maintain highly controlled growth conditions, with stable, high-quality lighting, and temperature and humidity control. Production conditions that influence marijuana yield and cannabinoid concentration include plant genotype and environmental conditions including temperature, water availability, and fertilizer application during the vegetative growth period, photoperiod, light type, and quality and the development stage of the plant (Lydon et al., 1987; Tipparat et al., 2012; Marti et al., 2014; Caplan et al., 2017). At a physiological level, plant growth regulators can also affect cannabinoid accumulation. For instance, application of gibberellic acid (GA_3_) can increase or decrease the accumulation of Δ_9_-tetrahydrocannabinol (THC) and cannabidiol (CBD) in cannabis leaves while abscisic acid (ABA) and cycocel increase THC content (Mansouri et al., 2011; Singh et al., 2011). The mechanism underlying these effects is not currently understood. One hypothesis is that the application of GA_3_ contributes to an increase of 1-aminocyclopropane-1-carboxylate (ACC), which subsequently increases ethylene levels in the plant. According to this theory, higher levels of ethylene result in increased THC and CBD contents (Mansouri et al., 2011).

In contrast, industrial/fiber hemp is grown outdoors, with a view to maximum biomass and yield at minimum production cost. Growing conditions, such as temperature, moisture, soil, seeding density, and photoperiod determine the yield and quality of hemp (Vogl et al., 2004; Hoppner and Mange-Hartmann, 2007; Townshend and Boleyn, 2008).

## Plant-Growth Promoting Rhizobacteria for Cannabis Production

Plant growth-promoting rhizobacteria are microbes associated with plant roots that promote plant growth by (1) providing enhanced mineral nutrition, (2) producing plant hormones or other molecules that stimulate plant growth and prime plant defenses against biotic and abiotic stresses, or (3) protecting plants against pathogens by affecting survival of pathogenic microorganisms (Podile and Kishore, 2006; Ortíz-Castro et al., 2009; Bhattacharyya and Jha, 2012; Nandal and hooda, 2013; Vacheron et al., 2013; Ahemad and Kibret, 2014; Yan et al., 2016; Rosier et al., 2018). PGPR are well-recognized as promising inputs for sustainable agricultural production (Bhattacharyya and Jha, 2012; Gupta et al., 2015; Backer et al., 2018).

PGPR-associated yield increases in other crops have been studied extensively. Many investigations have shown that PGPR strains can stimulate the growth of plants, including rice (Etesami et al., 2014), maize (Akladious and Abbas, 2012; Głodowska et al., 2016), soybean (Jayasinghearachchi and Seneviratne, 2004; Ramesh et al., 2014), and wheat (Dilfuza and Zulfiya, 2009). These yield increases have been associated with increased germination percentage (Gholami et al., 2011), seedling vigor (Bharathi et al., 2004), root and shoot growth, and total biomass production (van Loon et al., 1998).

### Yield and Quality Enhancements Associated With Plant-Growth Promoting Rhizobacteria

In the case of cannabis production, there is a lack of data about the use of PGPR due to past legal restrictions on production of this crop. There are only two publications (Conant et al., 2017; Pagnani et al., 2018) that report data regarding the benefits of PGPR inoculation on growth and yield of marijuana and hemp. Pagnani et al. (2018) showed that a consortium of PGPR (*Azospirillum brasilense*, *Gluconacetobacter diazotrophicus*, *Burkholderia ambifaria*, and *Herbaspirillum seropedicae*) improved the growth and physiological status of hemp plants and increased secondary metabolite accumulation and antioxidant activity. Conant et al. (2017) demonstrated that the microbial biostimulant product Mammoth P™ promoted hemp growth at the bloom stage but did not report effects on cannabinoid concentration. Previous studies have shown that PGPR inoculation alters secondary metabolite accumulation in other plant species (Kim et al., 2011; Vacheron et al., 2013; Braga et al., 2016; Mishra et al., 2018); this leads us to hypothesize that PGPR inoculation will alter cannabinoid levels/composition in cannabis. It is critical to determine the effect of PGPR on the yield of cannabis and on the biosynthesis and accumulation of cannabinoids, in particular, in plant tissues or organs at various growth stages.

Our laboratory has already illustrated that bacteria isolated from one plant species can trigger growth promotion and induce stress responses in other species, including crop plants (Smith et al., 2015; Fan et al., 2017; Ricci et al., 2019), which suggest that known PGPR may stimulate growth in cannabis. Moreover, these effects can be induced by inoculating a bacterium or a consortium of bacteria onto plants (Souza et al., 2015). We hypothesize that future research will demonstrate that PGPR-based inoculants can alter (1) cannabinoid accumulation, (2) increase flower yield for marijuana cultivars and seed and fiber yield for hemp cultivars, (3) protect against plant pathogens by production of antimicrobial compounds and priming of plant immune responses, and (4) reduce the impact of abiotic stresses associated with intensive indoor marijuana cultivation (e.g., salinity stress) and challenges associated with climate change, for outdoor hemp cultivation (e.g., drought, high temperatures, flooding).

### Biological Control and Disease Resistance Associated With Plant-Growth Promoting Rhizobacteria

Currently, PGPR species of the genera *Agrobacterium*, *Azospirillum*, *Azotobacter*, *Bacillus*, *Burkholderia*, *Delftia*, *Paenibacillus*, *Pantoea*, *Pseudomonas*, *Rhizobium,* and *Serratia* are used commercially as biocontrol agents (Glick, 2012). Some of them are already used in the production of various plants, to inhibit diseases *via* a range of mechanisms (Compant et al., 2005). For instance, *Pseudomonas fluorescens* controls downy mildew caused by *Sclerospora graminicola* of pearl millet (*Pennisetum glaucum*) (Raj et al., 2003) and *Bacillus* spp. can control bacterial leaf blight of rice caused by *Xanthomonas oryzae* (Udayashankar et al., 2011). Some *Pseudomonas* and *Bacillus* species are used as biological control agents against pests and plant diseases of potato (Hultberg et al., 2010) and sugar beet (Bargabus et al., 2004).

PGPR can help control plant pathogens by (1) direct antagonism against potential pathogens (Beneduzi et al., 2012), (2) competition for space and nutrients (Kumari and Srivastava, 1999), and/or (3) activating induced systemic resistance (ISR) in plants, to prevent infection by specific pathogens (Kloepper et al., 1980, 2004; van Loon et al., 1998; Jetiyanon and Kloepper, 2002; Van et al., 2009; Mishra et al., 2010; Egamberdieva et al., 2017). ISR is mediated by jasmonate (JA)- and ethylene (ET)-sensitive pathways (van Loon et al., 1998; Spoel and Dong, 2012). However, the ability of PGPR strains to elicit ISR appears to depend on the host/rhizobacterium combination (Beneduzi et al., 2012). When successfully activated by PGPR, ISR can enhance the defense capacity of plants by priming for potentiated expression of defense genes (Tjamos et al., 2005). It is clear that PGPR strains, inoculated onto plants, can increase the ability of plants to defend against specific pathogens by eliciting the production of endogenous plant hormones, such as IAA and GA_3_. Pieterse et al. (2000) found that following the induction of ISR, plants have an enhanced capacity to convert ACC to ethylene, which provides a greater potential to produce ethylene. However, Beneduzi et al. (2012) found that ET- and JA-dependent plant responses can be triggered without a concomitant increase these phytohormones, working instead by enhancing sensitivity to these hormones. Therefore, future research should attempt to determine if the application of PGPR can control infection of cannabis plants by pathogens due to ISR activation *via* production of plant hormones and/or increased expression of defense-related genes.

#### Powdery Mildew Control in Indoor Cannabis Cultivation: An Example of Potential Plant-Growth Promoting Rhizobacteria Application

Cannabis can be infected by a plethora of phytopathogens, leading to reduced plant productivity from the seedling to harvest stages (McPartland, 1996; Kusari et al., 2013). For example, *Botrytis cinerea* and *Trichothecium roseum* (McPartland, 1996) are commonly found on marijuana plants, especially outdoors, and can seriously damage the plant by attacking leaves, flowers, stems and branches. Indoor-produced cannabis plants are threatened by *Trichothecium roseum* (McPartland, 1991) and *Golovinomyces* sp. (Thompson et al., 2017), which attack the leaves and flowers, causing pink rot and powdery mildew diseases, respectively. It is highly desirable to effectively address these threats, to prevent yield losses in cannabis production.

Powdery mildew is a severe fungal disease that damages leaves and buds at all growth stages, and is especially common in indoor cannabis production, due to high humidity levels. Powdery mildew infection causes leaves to senescence prematurely affecting photosynthetic rate and yield, and reducing flower bud quality (McPartland, 1996; McPartland and Cubeta, 1997). Powdery mildew spores destroy the cannabis resin leading to reductions in the medicinal value of marijuana plants (McPartland, 1996). Thus, there is a significant need to develop effective methods to control powdery mildew in cannabis production.

Biological control of plant pathogens, including powdery mildew, provides several advantages over existing chemical control measures. To date, the application of chemical controls such as bicarbonates or refined horticultural oils, has been used to control powdery mildew in other crops (Fernandez et al., 2006). However, these sprays may injure young seedlings, and may have deleterious effects on soil structure (McPartland and Hillig, 2008). *Bacillus subtilis* has been shown to effectively control strawberry and cucurbit powdery mildew caused by *Sphaerotheca macularis* (Lowe et al., 2012) and *Podosphaera fusca* (García-Gutiérrez et al., 2013), respectively, while *Pseudomonas aeruginosa* can control pea powdery mildew when applied as a foliar spray (Bahadur et al., 2007). These results suggest that inoculating cannabis with PGPR may assist in controlling powdery mildew, representing a substantial advantage over currently available chemical control methods. In addition, fungicide residues could be eliminated on plant parts destined for human consumption (buds for marijuana and seeds for hemp) if an effective biocontrol technology could be applied as a root drench, instead of as a foliar spray.

## Examples of Widely Prevalent Phytomicrobiome Members: *Pseudomonas* and *Bacillus* for Growth Promotion and Disease Control in Cannabis

### Pseudomonas

In general, *Pseudomonas* spp. show good colonization in numerous ecological niches including soil, water, and plant surfaces (Parret et al., 2003; Humphris et al., 2005; Schreiter et al., 2018) and can inhibit the growth of plant pathogens and promote plant growth. *Pseudomonas* strains can promote plant growth by producing plant hormones such as IAA and ACC deaminase (Khan et al., 2016) and function as biocontrol agents by producing various pathogen-deterrent compounds, including antibiotics, polysaccharides and siderophores (Beneduzi et al., 2012; Santoyo et al., 2012; Souza et al., 2015). *Pseudomonas* can induce ISR and to date, experiments with *Pseudomonas* have concentrated on elucidating the molecular and physiological mechanisms that are the basis of ISR (Kloepper et al., 2004). Hultberg et al. (2010) demonstrated that strains of *Pseudomonas* can significantly reduce potato late blight disease caused by the oomycete *Phytophthora infestans*.

### Bacillus

*Bacillus* spp. promote plant growth by (1) excreting cytokinins into the rhizosphere (Arkhipova et al., 2005) and (2) stimulating the synthesis of phytohormones, such as IAA (Shao et al., 2015) and GA_3_ (Bottini et al., 2004; Idris et al., 2007). *Bacillus* spores act as biological control agents by inhibiting the growth of various pathogenic microbes (Emmert and Handelsman, 1999; Kumar et al., 2011). Studies have shown that the impact of *Bacillus* spp. varies among crop species and that the application of *Bacillus* can improve agronomic traits of crop plants and impart enhanced tolerance to some pathogens (Choudhary, 2011; Lyngwi and Joshi, 2013). Treatment with *Bacillus* spp. elicited ISR in most of the plant species evaluated and also altered secondary metabolite biosynthesis in plants; both effects contributed to protection against plant diseases (Kloepper et al., 2004). In contrast to *Pseudomonas*, using *Bacillus* strains to trigger the ISR pathway in plants is dependent on the ethylene and jasmonate pathways (Santoyo et al., 2012). To date, studies on *Bacillus* spp. as a biocontrol agents and elicitors of ISR have mainly focused on aspects of microbial ecology, the resilience of plants with activated ISR and direct plant growth promotion (Kloepper et al., 2004).

Overall, previous research has shown that these two PGPR genera have strong influences on plant growth promotion through the production of various substances (Table 1; Canbolat et al., 2006; Rajkumar et al., 2006; Wani et al., 2007; Poonguzhali et al., 2008; Rajkumar and Freitas, 2008; Tank and Saraf, 2009; Wani and Khan, 2010; Ma et al., 2011; Ahemad and Kibret, 2014), but their application remains virtually unexplored for cannabis production. Based on the work from our laboratory (Fan et al., 2018; Ricci et al., 2019), *Pseudomonas* and *Bacillus* are very common and often dominant bacteria associated with both cultivated and wild plants. Given the results of previous studies (Table 1), it would be very interesting to determine if any strains of these two extremely common PGPR strains have positive influences on cannabis yield and cannabinoid profiles. In addition, further studies should be conducted to investigate the mode-of-action of these two strains, to identify commonalities and unique mechanisms of growth promotion and biocontrol of plant pathogens.

**Table 1:** Plant growth-promoting substances released by *Pseudomonas* and *Bacillus*.

PGPR	Plant growth-promoting traits	References
*Pseudomonas* sp.	Production of ACC deaminase, IAA, siderophores, HCN, antibiotics; P solubilization; heavy metal chelation	Tank and Saraf (2009) Poonguzhali et al. (2008) Rajkumar and Freitas (2008)
*Pseudomonas* sp. A3R3	Production of IAA, siderophores	Ma et al. (2011)
*Bacillus* sp.	P solubilization	Canbolat et al. (2006)
*Bacillus* species PSB10	Production of IAA, siderophores, HCN, ammonia	Wani and Khan (2010)
*Pseudomonas* sp. *Bacillus* sp.	Production of IAA, siderophores, ammonia, HCN; antifungal activity; P solubilization	Rajkumar et al. (2006) Wani et al. (2007)

*ACC deaminase, 1-aminocyclopropane-1-carboxylate deaminase; IAA, indole-3-acetic acid; HCN, hydrogen cyanide*.

## Conclusions and Future Prospects

Cannabis is poised to become an important crop globally; its importance is increasing with the number of countries legalizing the use of cannabis for fiber production and medical applications. It is critical to investigate how to improve cannabis yields and alter cannabinoid concentration and composition. However, because cannabis use has been illegal in most of the world for the past century, there is a great shortage of reliable research data in this area.

The use of PGPR inoculants has contributed to improved yields for many other crops, as a result of nutrient mobilization, hormone production, disease control, and improved stress tolerance. Thus, study of the responses of cannabis to inoculation with PGPR could provide an efficient approach to improve cannabis yield and quality for medical use, and to do so in an environmentally sustainable way. PGPR also have the potential to provide an effective and acceptable strategy for control of key cannabis diseases, without the risks associated with pesticide residue. Overall, elements of the phytomicrobiome have the potential to increase the safety, yield and quality of cannabis.

## Author Contributions

DL gathered literature and prepared the manuscript. RB, WR, and DS provided feedback and oversaw progression of the manuscript. All authors gave final approval for publication and agreed to be held accountable for the work performed therein.

### Conflict of Interest Statement

WR is Chief Executive Officer of Ravenquest Biomed, Inc., a company that produces and sells cannabis products. DS conducts research in collaboration with this company, where the research is funded through the Natural Sciences and Engineering Research Council of Canada, which levers industrial funding.

The remaining authors declare that the research was conducted in the absence of any commercial or financial relationships that could be construed as a potential conflict of interest.
